# Sinomenine inhibits microglial activation by Aβ and confers neuroprotection

**DOI:** 10.1186/1742-2094-8-117

**Published:** 2011-09-14

**Authors:** Shilpa Mishra Shukla, Shiv K Sharma

**Affiliations:** 1 National Brain Research Centre, Manesar, Haryana-122050, India

## Abstract

**Background:**

Neuroinflammation is an important contributor to the development of neurodegenerative diseases, including Alzheimer's disease. Thus, there is a keen interest in identifying compounds, especially from herbal sources, that can inhibit neuroinflammation. Amyloid-β (Aβ) is a major component of the amyloid plaques present in the brains of Alzheimer's disease patients. Here, we examined whether sinomenine, present in a Chinese medicinal plant, prevents oligomeric Aβ-induced microglial activation and confers protection against neurotoxicity.

**Methods:**

Oligomeric amyloid-β was prepared from Aβ(1-42). Intracellular reactive oxygen species production was determined using the dye 2',7'-dichlorodihydrofluorescin diacetate. Nitric oxide level was assessed using the Griess reagent. Flow cytometry was used to examine the levels of inflammatory molecules. BV2-conditioned medium was used to treat hippocampal cell line (HT22) and primary hippocampal cells in indirect toxicity experiments. Toxicity was assessed using MTT reduction and TUNEL assays.

**Results:**

We found that sinomenine prevents the oligomeric Aβ-induced increase in levels of reactive oxygen species and nitric oxide in BV2 microglial cells. In addition, sinomenine reduces levels of Aβ-induced inflammatory molecules. Furthermore, sinomenine protects hippocampal HT22 cells as well as primary hippocampal cells from indirect toxicity mediated by Aβ-treated microglial cells, but has no effect on Aβ-induced direct toxicity to HT22 cells. Finally, we found that conditioned medium from Aβ-treated BV2 cells contains increased levels of nitric oxide and inflammatory molecules, but the levels of these molecules are reduced by sinomenine.

**Conclusions:**

Sinomenine prevents oligomeric Aβ-induced microglial activation, and confers protection against indirect neurotoxicity to hippocampal cells. These results raise the possibility that sinomenine may have therapeutic potential for the treatment of Alzheimer's diseases as well as other diseases that involve neuroinflammation.

## Background

Alzheimer's disease (AD) is a devastating neurodegenerative disorder that eventually leads to severe cognitive impairment. Although AD is typically a late onset disease, in a small number of familial cases it occurs early in life. Extracellular amyloid plaques and intracellular neurofibrillary tangles are the pathological hallmarks of AD. Amyloid-β (Aβ) is a major component of the plaques. Aβ is produced by processing of amyloid precursor protein, and plays important roles in the pathogenesis of AD. Aβ exists in several forms, including oligomeric forms. Oligomeric Aβ is thought to play an important role in the development of the disease [1,2]. Several studies have shown that oligomeric Aβ causes neuronal cell death, impairment in synaptic plasticity and memory deficits [e.g. [3-6]].

The available evidence suggests that neuroinflammation contributes to the development of neurodegenerative diseases, including AD [7,8]. Microglia are the resident immune cells in the brain. They are normally in a resting state, but they become activated in response to pathogens, toxins or cellular damage. Microglia are found in close association with the neuritic plaques in AD brain [9], and Aβ-induced inflammatory responses mediated by microglia are thought to contribute to neuronal toxicity [10]. Treatment of microglia with Aβ leads to release of inflammatory and toxic factors including reactive oxygen species (ROS) and nitric oxide (NO) [11,12], which may lead to neuronal cell damage and eventual death. Aβ inhibits long-term potentiation (LTP), which is considered a promising cellular mechanism for memory formation. Importantly, inhibition of LTP by Aβ also involves microglia [13]. Thus, in addition to direct neuronal cell death, Aβ causes indirect neuronal cell death due to neuroinflammation, and inhibits synaptic plasticity.

Considering the available supporting literature regarding the role of microglial activation in neurodegenerative disorders, there is keen interest in identifying compounds from natural sources that can reduce or prevent neuroinflammation, and which thus could be beneficial in neurodegenerative diseases, including AD. Sinomenine is an alkaloid isolated from *Sinomenium acutum*, a Chinese medicinal plant. It is a dextrorotatory morphinan analog which shares structural similarity with morphine, and weakly binds to the opioid μ-receptor [14]. Qian and colleagues [15] have shown that sinomenine protects dopaminergic neurons against lipopolysaccharide (LPS)-induced cell death in neuron-glia cultures. NADPH oxidase (PHOX) activity is involved in the protective effects of sinomenine. In addition, this compound confers protection against 1-methyl-4-phenylpyridinium (MPP+)-induced cell death. Wang and colleagues [16] found that sinomenine reduces advanced glycation end products-induced increases in the levels of cytokines in retinal microglial cells. Furthermore, this compound shows beneficial effects in rheumatoid arthritis and mesangial proliferative nephritis [17], inhibits morphine withdrawal symptoms [18], and shows protective effects against cold ischemia/reperfusion injury [19]. In this study, we have examined the effects of sinomenine on oligomeric Aβ-induced microglial activation. In addition, we have investigated the protective effects of this compound on neuronal toxicity caused by Aβ.

## Methods

### Preparation of oligomeric amyloid beta

Oligomeric amyloid-β (Aβ-derived diffusible ligands, ADDL) was prepared using amyloid-β 1-42 peptide (American Peptide) as described previously [20] with minor modifications. The peptide was dissolved in 1,1,1,3,3,3-Hexafluoro-2-propanol (HFIP, Fluka), aliquoted, dried in fume hood and stored at -80°C. The peptide film was dissolved in DMSO to 5 mM concentration and further diluted in phosphate-buffered saline (PBS) to make a 100 μM solution. This preparation was incubated at 4°C for 24 h. To remove insoluble material, the preparation was centrifuged at 14,000 g for 10 min at 4°C. The soluble fraction (ADDL) was stored at -80°C until use. Protein concentration was determined using BCA reagent with bovine serum albumin as standard. ADDL was used at a 2 μM final concentration [21].

### Cell culture and treatments

Sinomenine (Sigma-Aldrich) was dissolved in DMSO and diluted to different concentrations in DMEM such that the final DMSO concentration was 0.1%. BV2 microglial cells were obtained from Dr. A. Basu of our Centre and cultured in DMEM with 10% fetal bovine serum (FBS). The cells were serum starved for 4-8 h before treatment. The control cultures received vehicle for ADDL. For the analysis of reactive oxygen species and nitric oxide, and for assaying the levels of inflammatory molecules described under "sinomenine reduces A-beta-induced increases in inflammatory molecules", cells were cultured in 24-well plates (3.5 × 10^4^cells per well). After serum starvation, BV2 cells were treated with sinomenine for 1.5 h, then with ADDL for 12 h, co-incident with ADDL treatment of a sister culture. For "pre-treatment condition" ADDL and sinomenine treatment was done as described above, whereas for "simultaneous addition", ADDL and sinomenine were added to the culture at the same time. After treatment, samples were used for different assays. For the indirect toxicity experiments and for the analysis of NO and inflammatory molecules in the BV2 conditioned media, BV2 cells grown in 24-well plates (3 × 10^4 ^cells per well) were treated with sinomenine for 1.5 h, then with ADDL for 6 h, co-incident with ADDL treatment of a sister culture. After treatment, the cells were washed and fresh medium was added without ADDL or sinomenine. The conditioned medium was collected after a 12 h period and then centrifuged to obtain cell-free supernatant. In all cases sinomenine was present throughout ADDL treatment. Where sinomenine alone was used, the cultures were treated with sinomenine (without ADDL) similar to the sinomenine + ADDL condition.

Hippocampal HT22 cells were a kind gift from Dr. D. Schubert, The Salk Institute, La Jolla, California. The cells were cultured in DMEM with 10% FBS [in a 96-well plate (5 × 10^3 ^cells per well) for MTT assay or in poly-D-lysine-coated 4-well chamber slide (1 × 10^4 ^cells per well) for TUNEL assay]. For the indirect toxicity experiments, HT22 cells were serum-starved for 4 h and then treated with a mixture of 50% BV2-conditioned medium and 50% fresh DMEM. For MTT assay, cells were treated for 44 h (before addition of MTT), and for the TUNEL assay, cells were treated for 48 h. For direct toxicity experiments HT22 cells were serum-starved for 2.5 h, treated with sinomenine for 1.5 h, then treated with ADDL, co-incident with ADDL treatment of a sister culture. Sinomenine was present throughout the ADDL treatment. ADDL treatment was for 20 h (before addition of MTT) for MTT assay and 24 h for TUNEL assay.

For primary hippocampal cultures, Sprague Dawley pregnant female rats were sacrificed according to a protocol approved by the Institutional Animal Ethics Committee and hippocampal cultures were prepared from E18-E20 embryos as described previously [21] with minor modifications. Briefly, hippocampi were isolated and triturated to obtain dissociated cells which were then seeded in 90 mm dishes in DME\F12 medium with 10% FBS. After 16-20 h, the medium was replaced with Neurobasal medium containing B27 supplement, glutamax (all from Invitrogen) and glutamic acid (Sigma-Aldrich). After 4 days in vitro (DIV), cells were detached from the plates and seeded in 8-well chamber slides (6 × 10^4 ^cells per well) in 50% fresh Neurobasal maintenance medium (Neurobasal medium containing B27 supplement and glutamax) mixed with 50% neuronal conditioned medium. Ara C (5 μM; Sigma-Aldrich) was added to reduce glial cell proliferation. Fifty percent of the medium was replaced every 2 days with Neurobasal maintenance medium, and cultures were used for treatment on DIV 8-9. For indirect toxicity experiments, cells were treated for 24 h with 50% BV2-conditioned medium that was mixed with 50% fresh Neurobasal maintenance medium.

### Reactive oxygen species assay

BV2 cells were treated under different conditions, and the level of intracellular ROS was measured fluorimetrically using the dye 2',7'-dichlorodihydrofluorescin diacetate (DCFDA; Sigma-Aldrich) as described previously [21,22]. The cells were incubated with DMEM containing 5 μM DCFDA for 1 h at 37°C, washed with PBS and lysed in lysis buffer (10 mM Tris pH 7.9, 150 mM NaCl, 1 mM EDTA, 0.2 mM EGTA, 0.2 mM NaVO3, 0.5% NP-40 and 1% Triton X-100). The lysate was centrifuged at 10,000 g for 15 min. A 10-μl aliquot of supernatant was mixed with 90 μl of PBS in a 96-well black plate and fluorescence was measured using a Varioskan Flash multimode Reader (Thermo Electron Corporation, Finland) at an excitation wavelength of 485 nm and an emission wavelength of 530 nm. The readings obtained were normalized with the amount of protein in each sample. Data are expressed as a percentage of control cultures.

### Nitric oxide assay

After different treatments of BV2 cells, released nitric oxide was measured in the culture medium using Griess reagent (Sigma-Aldrich). A 100-μl aliquot of cell-free culture medium was incubated with 100 μl of Griess reagent in the dark at room temperature for 15 min. The intensity of color developed was measured at 540 nm using a Benchmark Plus 96-well ELISA plate Reader (BioRad). Data are expressed as a percentage of control samples.

### Cytokine bead array assay

BV2 cells were treated under different conditions, and the levels of inflammatory molecules were measured in cell free culture medium using a Mouse Inflammation cytokine bead array kit (Becton Dickinson) as described previously [23] with minor modifications. Briefly, a 30-μl bead mix was incubated with an aliquot of cell-free culture medium and 30 μl of phycoerythrin detection reagent for 2 h at room temperature in the dark. The beads were then washed with wash buffer (provided with the kit), re-suspended in 300 μl of the wash buffer and analyzed in FACS Calibur using Cell Quest Pro Software and BD CBA software (Becton Dickinson, San Diego, CA). The standard curve was prepared according to the kit's manual. Data are expressed as fold relative to control.

### MTT assay

Cell viability of HT22 cells was assessed using 3-(4,5-dimethyl-2-thiazolyl)-2,5-diphenyl-2H-tetrazolium bromide (MTT, Sigma-Aldrich) assay. After treatment, MTT reagent was added to the wells, incubated for 4 h, and the samples were processed for MTT assay as described previously [21]. The absorbance was measured at 570 nm. The mean of readings of triplicate wells was taken as one value. The OD value for the control cultures was considered as 100% viability and viability in other samples is expressed as a percentage of viability in the control cultures.

### Terminal deoxynucleotidyl transferase-mediated dUTP nick-end labeling (TUNEL) assay

After treatments, cells were fixed and processed for TUNEL assay as described previously [21]. The total number of DAPI (4'6 diamidino-2-phenylindole)-stained or TUNEL-positive cells, in 5 different frames, were counted. The average number of cells (DAPI-stained) per frame in experiments ranged from 162-213.6 (control), 170.8-215.6 (ADDL) and 175.2-217 (ADDL + sinomenine) in HT22 indirect toxicity experiments, 123.4-130.2 (control), 124-135.2 (ADDL) and 125.8-134.4 (ADDL + sinomenine) in primary hippocampal cell indirect toxicity experiments, and 87.4-121 (control), 81-105.6 (ADDL) and 82.2-101.8 (ADDL + sinomenine) in HT22 direct toxicity experiments. Data are expressed as percent TUNEL-positive cells.

### Data analysis

Data were analyzed using a paired Student's t-test. Differences were considered significant when the p value was less than 0.05. Data are expressed as mean ± SEM.

## Results

### Sinomenine inhibits amyloid-β-induced increase in level of reactive oxygen species in microglial cells

**Figure 1:**
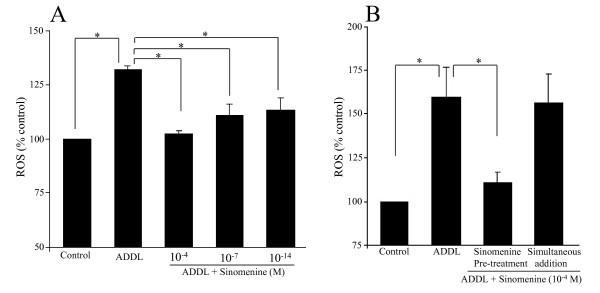
**Pre-treatment, but not simultaneous treatment, of sinomenine inhibits oligomeric Aβ (ADDL)-induced increase in level of reactive oxygen species (ROS) in microglial BV2 cells**. ROS levels were determined using the DCFDA reagent. A. BV2 cells were pre-treated with different concentrations of sinomenine before addition of ADDL. ADDL significantly increased the level of ROS in BV2 cells. However, pre-treatment with sinomenine inhibited ADDL-induced increase in ROS level (n = 6 in all groups). B. BV2 cells were either pre-treated with sinomenine before ADDL addition (Sinomenine pre-treatment) or treated with sinomenine and ADDL simultaneously (Simultaneous addition). Whereas pre-treatment of sinomenine inhibited ADDL-induced ROS generation, simultaneous treatment of sinomenine had no effect on ADDL-induced ROS level (n = 7 in all groups). There was no significant difference between ADDL and ADDL + sinomenine groups when simultaneous addition was performed. In Figures 1A, 2, 5, 6 and 8, the effects of sinomenine alone were also examined. Compared to control, sinomenine alone had no significant effects. Asterisks denote significant differences (p < 0.05).

Previous studies have shown that treatment of microglial cells with Aβ increases the level of reactive oxygen species (ROS) [e.g. 24]. Using DCFDA, a commonly used reagent to measure intracellular ROS [21,22], we found that treatment of BV2 cells with oligomeric Aβ induced a significant increase in the level of ROS [135.8% ± 2.56 (% control)]. We next examined whether sinomenine has any effect on the level of ROS induced by oligomeric Aβ. For this analysis, we treated BV2 cells with ADDL or ADDL plus different concentrations of sinomenine. Taking a clue from previous studies [15,16], we used 10^-14 ^M, 10^-7 ^M and 10^-4 ^M sinomenine in our experiments. We found that whereas ADDL treatment increased the level of ROS, sinomenine decreased ROS level induced by oligomeric Aβ (Figure 1A). Sinomenine alone at all three concentrations had no significant effect on basal ROS levels (data not shown). This observation is consistent with that of Wang et al [16] who found that sinomenine does not affect the basal level of ROS in microglial cells. All sinomenine concentrations tested reduced ADDL-induced increase in ROS generation, but the 10^-4 ^M concentration gave the best results. Hence, in subsequent experiments, we used this concentration of sinomenine.

In these experiments, BV2 cells were treated with sinomenine before addition of ADDL (Pre-treatment condition). Thus, we next asked whether simultaneous treatment of sinomenine and ADDL has any effect on ADDL-induced ROS generation. We found that whereas pre-treatment with sinomenine inhibited ADDL-induced ROS generation, simultaneous treatment with sinomenine did not reduce ADDL-induced ROS level (Figure 1B). Thus, pretreatment with sinomenine is required for its effect on ADDL-induced ROS level. In subsequent experiments, pretreatment with sinomenine was used to examine its effects in different assays.

### Sinomenine inhibits amyloid-β-induced increase in level of nitric oxide in BV2 cells

**Figure 2:**
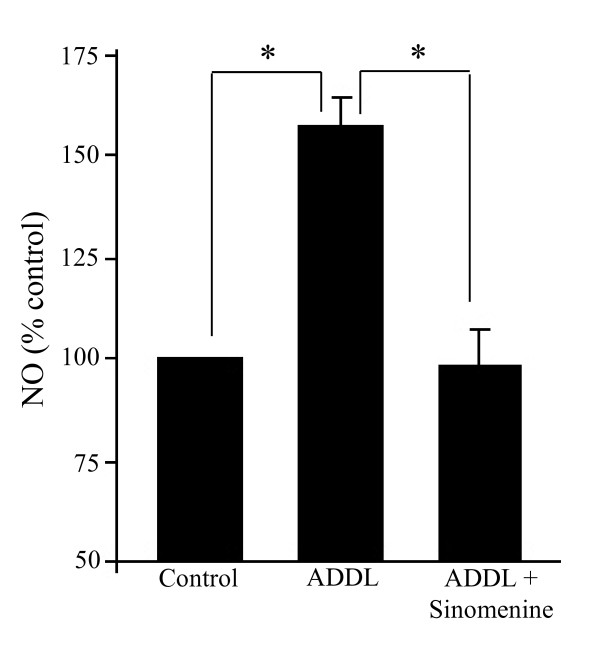
**Sinomenine inhibits Aβ-induced increase in the level of nitric oxide (NO) in BV2 cells**. BV2 cells were treated with oligomeric Aβ (ADDL) in the presence or absence of sinomenine. NO release was estimated using the Griess reagent. ADDL treatment of BV2 cells increased the level of NO, but sinomenine inhibited the effect of ADDL on NO level (n = 7 in all groups). Asterisks denote significant differences (p < 0.05).

Aβ is known to increase levels of inducible nitric oxide synthase (iNOS) in microglial cells [25,26]. Since the induction of iNOS is associated with increased production of nitric oxide, we next examined whether sinomenine has any effect on the production of NO. The level of NO was measured indirectly by the amount of nitrite present in the culture medium [26]. Consistent with previous studies [27,28], we found that treatment of BV2 cells with ADDL led to a significant increase in the level of NO. However, sinomenine reduced NO level (Figure 2). Thus, sinomenine inhibits ADDL-induced enhancement of NO level in BV2 cells.

**Figure 3:**
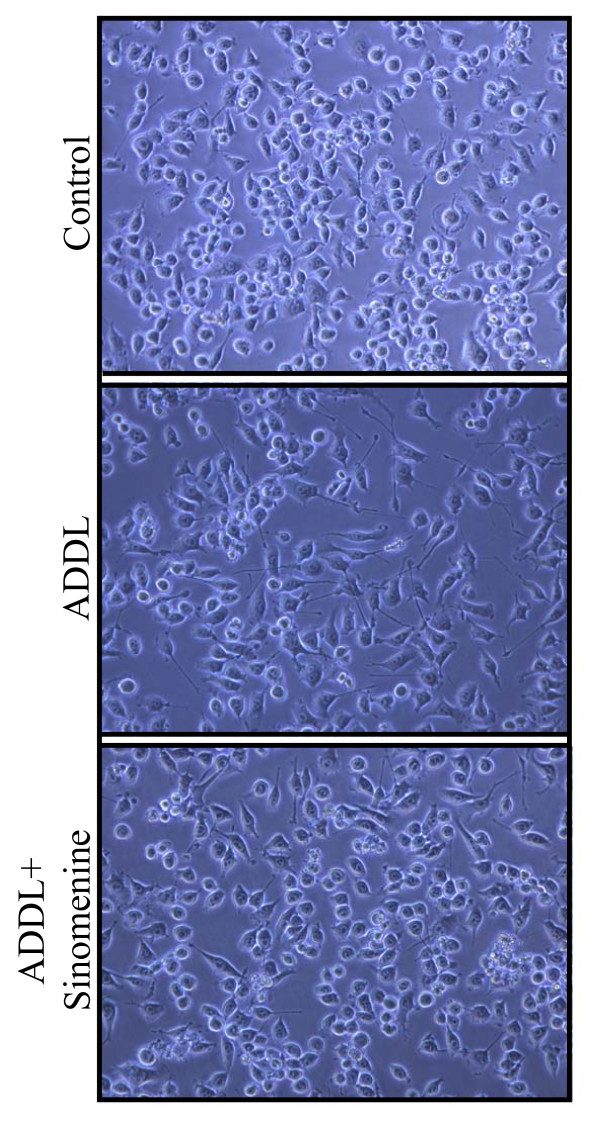
**Sinomenine inhibits morphological changes induced by oligomeric amyloid-β**. Phase contrast images (20× magnification) of BV2 cells treated with oligomeric Aβ (ADDL) in the presence or absence of sinomenine show that ADDL treatment led to extended processes and elongated morphology of the cells, but sinomenine reduced these ADDL-induced morphological changes.

The cellular morphology of some of the cultures used for ROS and NO assays was also examined. ADDL-treated BV2 cells showed more extended processes with elongated morphology. Sinomenine reduced the effects of ADDL on morphological changes in BV2 cells (Figure 3).

### Sinomenine reduces A-beta-induced increases in inflammatory molecules

**Figure 4:**
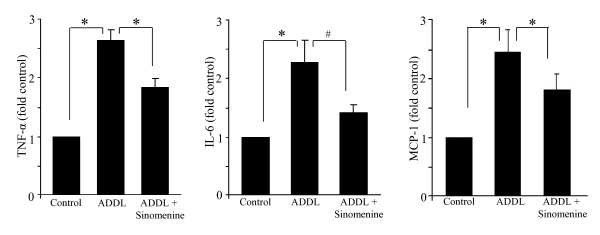
**Sinomenine reduces Aβ-induced increases in inflammatory molecules**. BV2 cells were treated with oligomeric Aβ (ADDL) in the presence or absence of sinomenine and levels of inflammatory molecules were determined using flow cytometry. Treatment with ADDL led to an increase in levels of TNF-α, IL-6 and MCP-1. However, treatment with sinomenine decreased levels of inflammatory molecules (n = 7 for TNF-α and IL-6, n = 4 for MCP-1). *, p < 0.05 and #, p < 0.061.

Treatment of microglial cells with Aβ has previously been shown to increase the levels of inflammatory molecules [26,29,30]. Thus, we examined whether sinomenine has any effects on oligomeric Aβ-induced release of cytokines and chemokine from BV2 cells. Cell-free culture medium, after treatment of BV2 cells with ADDL for 12 h in the presence or absence of sinomenine, was used to assay levels of IL-6, IL-10, IL-12, TNF-α, IFN-γ and MCP-1. Statistically significant increases in levels of IL-6, TNF-α, MCP-1 and IL-12 were observed following treatment with ADDL. Treatment with sinomenine reduced the levels of TNF-α and MCP-1 (Figure 4). Sinomenine reduced the level of ADDL-induced IL-6, although this was not statistically significant (p < 0.061). Although sinomenine was effective in reducing ADDL-induced increases in levels of inflammatory molecules, the levels of these molecules were still more than the levels in control cultures. These results are consistent with the findings of Qian and colleagues [15] who found that sinomenine did not completely block LPS-induced increases in TNF-α in microglial cells. Sinomenine did not affect ADDL-induced increase in level of IL-12 (fold control, ADDL = 1.39 ± 0.15; ADDL + sinomenine = 1.27 ± 0.11, p > 0.26 compared to ADDL; n = 7 in all groups). ADDL did not significantly affect the levels of IFN-γ or IL-10, and sinomenine did not affect the levels of these molecules (fold control, IFN-γ, ADDL = 1.1 ± 0.16, p > 0.6 compared to control; ADDL + sinomenine = 1.16 ± 0.08, p > 0.43 compared to ADDL; IL-10, ADDL = 1.28 ± 0.2, p > 0.30 compared to control; ADDL + sinomenine = 1.16 ± 0.12, p > 0.45 compared to ADDL; n = 7 in all groups). Collectively, these results show that sinomenine reduces oligomeric Aβ-induced release of inflammatory and toxic substances.

### Sinomenine confers protection to hippocampal HT22 cells against indirect toxicity

**Figure 5:**
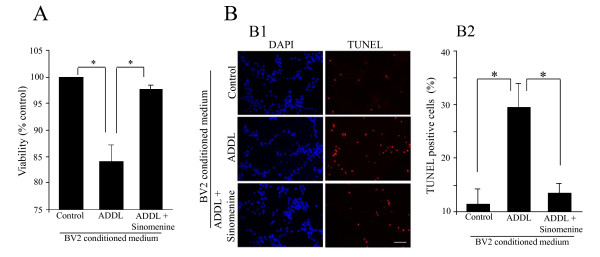
**Sinomenine inhibits Aβ-induced indirect toxicity to HT22 cells**. A. An MTT reduction assay shows that conditioned medium from ADDL-treated BV2 cells reduces the viability of HT22 cells. However, the decrease in cell viability was ameliorated by sinomenine (n = 4 in all groups). B. TUNEL assay shows that sinomenine prevents indirect toxicity to HT22 cells. Sample images (B1) and quantified summary data (B2) of DAPI- or TUNEL-stained HT22 cells treated with different conditioned media show that conditioned medium from ADDL-treated BV2 cells significantly increased the number of TUNEL-stained cells. However, conditioned medium from ADDL + sinomenine-treated BV2 cells did not increase the number of TUNEL positive cells (n = 4 in all groups). Asterisks denote significant differences (p < 0.05). Scale bar, 100 μm.

As noted earlier, activated microglial cells release substances that can cause toxicity to neurons. This indirect toxicity could also play important roles in the development of neurodegenerative diseases including AD. Since we found that sinomenine inhibits ADDL-induced production of inflammatory and toxic molecules, we next asked whether it affects indirect toxicity to hippocampal cells. For this purpose, we used a hippocampal cell line, HT22 that has been used in previous studies to examine toxicity by different agents, including Aβ [31-34]. We first used an MTT reduction assay to examine the effect of sinomenine on ADDL-induced indirect toxicity to HT22 cells. We found that when HT22 cells were treated with conditioned medium from ADDL-treated BV2 cells, there was a significant decrease in cell viability. However, when the cells were treated with conditioned medium from BV2 cells treated with ADDL and sinomenine, the cell viability was close to that of control cultures (Figure 5A). Thus, sinomenine protects HT22 cells against indirect toxicity induced by ADDL.

We used another measure, TUNEL assay, to examine the protective effect of sinomenine against indirect neurotoxicity. This assay is based on labeling of fragmented DNA during cell death. In this assay also, we found that treatment of HT22 cells with conditioned medium from ADDL-treated BV2 cells led to significant toxicity as evident by increased number of cells that were positive for TUNEL staining. In contrast, conditioned medium from ADDL plus sinomenine-treated BV2 cells did not increase the number of TUNEL-positive cells (Figure 5B). These results suggest that the neuronal toxicity was mediated by factors released from the microglial cells after treatment with ADDL, and that sinomenine confers protection to HT22 cells against indirect toxicity by oligomeric Aβ.

### Sinomenine confers protection to primary hippocampal cells against indirect toxicity

**Figure 6:**
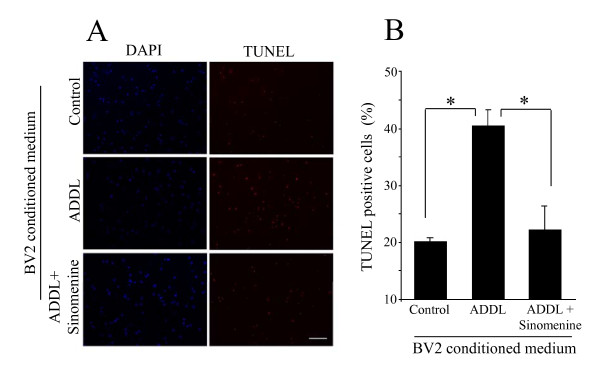
**Sinomenine inhibits Aβ-induced indirect toxicity to primary hippocampal cells**. (A) Sample images of DAPI- or TUNEL-stained primary hippocampal cells treated with BV2-conditioned media as indicated. Scale bar, 100 μm. (B) Quantified summary data show that conditioned medium from ADDL-treated BV2 cells significantly increased the number of TUNEL-positive cells. However, the number of TUNEL-positive cells was not increased when the cells were treated with conditioned medium from ADDL plus sinomenine-treated BV2 cells (n = 3 in all groups). Asterisks denote significant differences (p < 0.05).

Having shown that sinomenine protects HT22 cells against indirect toxicity induced by Aβ, we next asked whether it has any effect on indirect toxicity to primary hippocampal cells. For these experiments, we again used TUNEL staining. We found that treatment of primary hippocampal cells with conditioned medium from ADDL-treated BV2 cells led to a significant increase in the number of TUNEL-positive cells. However, treatment with conditioned medium from ADDL plus sinomenine-treated BV2 cells showed reduced number of TUNEL-positive cells (Figure 6). Thus, sinomenine also protects primary hippocampal cells from indirect toxicity by oligomeric Aβ.

**Figure 7:**
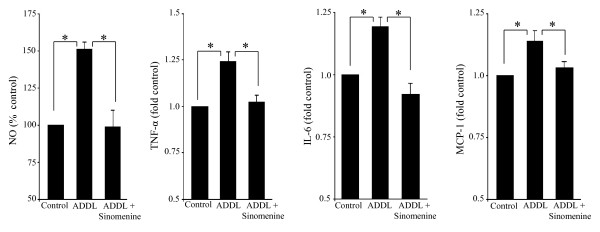
**Levels of nitric oxide and inflammatory molecules in BV2-conditioned media**. The levels of nitric oxide (NO) and inflammatory molecules in BV2-conditioned media prepared similarly to the conditioned media used for indirect toxicity experiments, were assayed using Griess reagent and flow cytometry, respectively. Oligomeric Aβ (ADDL) increased levels of NO (n = 4 in all groups), TNF-α, IL-6 and MCP-1 (n = 8 in all groups) in BV2-conditioned medium, but sinomenine reduced the levels of these molecules. Asterisks denote significant differences (p < 0.05).

Since the conditioned medium of BV2 cells treated with oligomeric Aβ was toxic to HT22 and primary hippocampal cells, it was of interest to determine if the conditioned medium contained higher levels of toxic molecules, and whether the levels of these molecules were affected by sinomenine. For these experiments, BV2 cells were treated with oligomeric Aβ for 6 h with or without sinomenine. The medium was then replaced with fresh medium without Aβ and sinomenine, and incubation was carried out for another 12 h (the same conditions as used for the collection of conditioned media for the indirect neurotoxicity experiments described above). The conditioned media were then assayed for NO and inflammatory molecules. We found increased levels of NO, IL-6, TNF-α and MCP-1 in the ADDL-treated BV2-conditioned medium. However, the levels of these molecules were reduced in conditioned medium from BV2 cells treated with ADDL and sinomenine (Figure 7). ADDL did not significantly affect levels of IFN-γ, IL-10 and IL-12; and sinomenine did not affect the level of these molecules (fold control, IFN-γ, ADDL = 1.25 ± 0.29, p > 0.52 compared to control; ADDL + sinomenine = 1.13 ± 0.15, p > 0.61 compared to ADDL; IL-10, ADDL = 1.22 ± 0.15, p > 0.27 compared to control; ADDL + sinomenine = 1.10 ± 0.10, p > 0.54 compared to ADDL; IL-12, ADDL = 1.13 ± 0.13, p > 0.55 compared to control; ADDL + sinomenine = 1.03 ± 0.13, p > 0.32 compared to ADDL, n = 8 in all groups). These results show that ADDL increased levels of NO and inflammatory molecules in BV2-conditioned medium, but sinomenine reduced their levels.

### Sinomenine does not confer protection to hippocampal cells against direct toxicity

**Figure 8:**
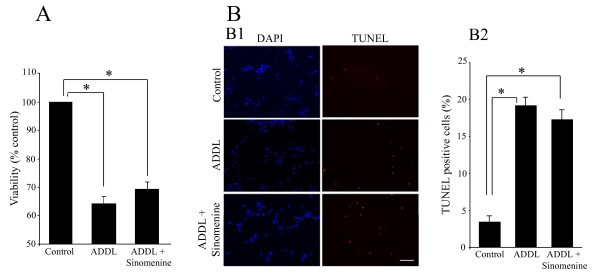
**Sinomenine does not confer protection against oligomeric Aβ-induced direct toxicity to HT22 cells**. A. An MTT reduction assay shows that ADDL treatment of HT22 cells reduced cell viability. The decrease in cell viability was not affected by sinomenine (n = 6 in all groups). There was no significant difference between ADDL and ADDL + sinomenine groups. B. TUNEL assay shows that sinomenine does not prevent ADDL-induced direct toxicity to HT22 cells. Sample images (B1) and quantified summary data (B2) of DAPI- or TUNEL-stained HT22 cells treated with different reagents show that ADDL treatment significantly increased the number of TUNEL-positive cells. The increase in the number of TUNEL-positive cells was not affected by sinomenine treatment (n = 3 in all groups). There was no significant difference between ADDL and ADDL + sinomenine groups. Scale bar, 100 μm. Asterisks denote significant differences (p < 0.05).

Having shown that sinomenine protects hippocampal cells against ADDL-induced indirect toxicity, we next asked whether this compound has any protective effect against direct A-beta toxicity. We found that treatment of HT22 cells with ADDL led to significant reduction in viability as assessed by MTT reduction assay. However, sinomenine did not affect ADDL-induced reduction in HT22 cell viability (Figure 8A). In the TUNEL assay also, we found that ADDL treatment increased the number of TUNEL-positive cells, and that this effect was not affected by sinomenine (Figure 8B). Thus, while sinomenine has protective effects against indirect neurotoxicity induced by A-beta, it does not show protection against direct toxicity.

## Discussion

In this study, we show that sinomenine, an alkaloid from a Chinese medicinal plant, inhibits oligomeric Aβ-induced increases in levels of ROS, NO and inflammatory molecules. In addition, sinomenine confers protection to hippocampal cells (HT22) against indirect toxicity. Furthermore, sinomenine also protects primary hippocampal cells from indirect toxicity.

Considerable evidence points to an important role for Aβ in the pathogenesis of AD. With regards to neurotoxicity, Aβ can directly cause neuronal cell death (direct toxicity) or Aβ can affect microglial cells to produce inflammatory and toxic factors that then affect the viability of neurons. The second mode of neuronal toxicity is referred to as indirect toxicity. Both kinds of toxicity mechanisms have been described in the literature. Microglia are the brain's resident immune cells that offer defense against pathogens. These cells are associated with the amyloid plaques in the brains of both human AD patients and AD transgenic animals [10]. Microglia-mediated inflammation has been implicated in the pathogenesis of neurodegenerative disorders including AD [11,35]. Thus, while microglial function is important for normal functioning of the brain, over-activation of microglia could have deleterious effects [10,12].

Aβ induces ROS generation in microglial cells [36]. In addition, Aβ induces the production of NO in these cells [26-28]. Reactive oxygen species and nitric oxide have been implicated in the pathogenesis of AD. The analysis of AD brain samples reveals evidence of ROS and NO production [10]. In addition, Aβ activates microglia leading to the release of inflammatory molecules [26]. An enhancement in cytokine levels is observed in AD transgenic animals [37,38]. Collectively, several studies suggest that Aβ activates microglia which may contribute to AD pathology by promoting inflammation and neuronal toxicity. We found that sinomenine inhibits ADDL-induced production of ROS, NO and inflammatory molecules. Importantly, we also showed that sinomenine confers protection against indirect toxicity to hippocampal cells. Our results are consistent with the study of Wang et al [16], who showed that sinomenine inhibits advanced glycation end products-induced release of cytokines, and enhancement of ROS production, in retinal microglial cells. In addition, Qian and colleagues showed that sinomenine inhibits LPS-induced NO and ROS production [15]. These authors showed also that sinomenine confers protection to dopaminergic neurons against LPS- and MPP+-induced toxicity in neuron-glia cultures.

Although sinomenine was effective in reducing indirect toxicity, it did not confer protection to hippocampal HT22 cells in direct toxicity experiments. This finding is consistent with that of Qian et al [15] who showed that although sinomenine protects dopaminergic neurons against MPP+-induced toxicity in neuron-glia cultures, it has no effects in neuron-enriched cultures.

Since inhibition of microglia-mediated damage could be helpful in at least delaying the progression of AD, anti-inflammatory therapy is considered a promising strategy in this disease. It has been shown that intraperitoneally injected or orally administered sinomenine can reach the brain [39,40] suggesting that it can cross blood-brain barrier. In addition, intraperitoneally injected sinomenine confers protection in ischemic brain injury [41]. It would be interesting to examine whether sinomenine reduces inflammation and confers neuroprotection *in vivo *in a model of Alzheimer's disease. Since sinomenine does not confer protection against Aβ-induced direct neuronal toxicity, the protection observed would likely be due to its effects on microglia. Our results, along with those of other studies, suggest that sinomenine may have therapeutic potential in neurodegenerative diseases that involve neuroinflammation.

## Conclusions

Our results show that sinomenine inhibits oligomeric amyloid-β-induced increases in levels of ROS, NO and inflammatory molecules in BV2 microglial cells. Moreover, this compound protects immortalized as well as primary hippocampal cells from indirect toxicity mediated by amyloid-β-treated BV2 cells. Thus, sinomenine may have therapeutic value in neurodegenerative diseases, including Alzheimer's disease.

## Competing interests

The authors declare that they have no competing interests.

## Authors' contributions

SMS and SKS conceived the study and designed the experiments. SMS performed the experiments. Both analysed the data and wrote the paper. Both authors have read and approved the final manuscript.
